# Frontotemporal Dementia and Suicide; Could Genetics be a Key Factor?

**DOI:** 10.1177/1533317520925982

**Published:** 2020-06-10

**Authors:** Rachel Fremont, Jordan Grafman, Edward D. Huey

**Affiliations:** 1 Department of Psychiatry, Columbia University, College of Physicians and Surgeons and New York State Psychiatric Institute, NY, USA; 2 Brain Injury Research Program, Rehabilitation Institute of Chicago, IL, USA; 3 Department of Physical Medicine and Rehabilitation, Feinberg School of Medicine, Northwestern University, Chicago, IL, USA; 4 Taub Institute and Gertrude H. Sergievsky Center, Columbia University Medical Center, New York, NY, USA; 5 Department of Neurology, Columbia University, College of Physicians and Surgeons, New York, NY, USA

**Keywords:** frontotemporal dementia, suicide, neuropsychiatry, dementia

Frontotemporal dementia (FTD) is a neurodegenerative syndrome characterized by degenerative changes in behavior, executive function, and/or language that are accompanied by volume loss in frontal and anterior temporal brain regions.^1^ Neuropsychiatric symptoms are a prominent feature of this disorder and can include apathy, behavioral disinhibition, loss of empathy, compulsive behaviors, and others.^2^ Overall frequency of formal current or past psychiatric disorder diagnosis is not thought to be increased in patients with FTD^3^ although this is somewhat controversial.^4^ Regarding depression, a meta-analysis in 2015 exploring the prevalence of depressive symptoms in FTD found that depressed mood is likely elevated in patients compared to healthy age matched controls and as prevalent in FTD as in other dementias including Alzheimer disease, vascular dementia, and dementia with Lewy bodies.^5^ Also, it is known that patients with dementia have a 3 to 10-fold increased risk of death by suicide.^6^ However, there have been few studies examining the prevalence of suicidality in FTD patients specifically.

Addressing this knowledge gap, a recent paper^7^ reported that patients with behavioral variant frontotemporal dementia (bvFTD) have significantly elevated suicidal ideation and suicide attempts when compared to healthy age and education matched controls. An earlier 2014 retrospective study of suicidal behavior in FTD patients similarly reported that suicidal behaviors were increased in FTD patients when compared to age and gender matched controls.^8^ However, both of these studies focused on patients with significant dementia and did not explore whether genetics might play a role in suicidality of FTD patients.

We wondered whether a similar increase in suicidality would be present in early/prodromal FTD patients with *MAPT* mutations that result in behavioral variant (bv)FTD with a high prevalence. We performed structured clinical psychiatric interviews^9^ on 12 prodromal *MAPT* mutation carriers, CDR = 0 to 0.5, and 46 control nonmutation carriers from the same families.^10^ We found that 8% of MAPT mutation carriers (1/12) had suicidal ideation in their lifetime, whereas 15% of control nonmutation carriers (7/46) from the same families had a lifetime history of suicidal ideation; 8% of MAPT mutation carriers (1/12) and 7% of control nonmutation carriers (3/46) reported current suicidal ideation. We were also interested in examining suicidality in patients who met full criteria for FTD^11^ in an independent and North American sample and so we examined suicidality measures on the Personality Assessment Inventory^12^ in 21 patients with sporadic FTD (n = 18 bvFTD and n = 3 primary progressive aphasia) enrolled in an ongoing study at National Institutes of Health / National Institute of Neurological Disorders and Stroke (NIH/NINDS). These participants had mild to moderate FTD (mean Mattis Dementia Rating Scale 2^13^ scores were 123 with a standard deviation of 10 for the bvFTD, and 110 with a standard deviation of 17 for the primary progressive aphasia participants). For both studies, informed consent was obtained by the participant or an appointed surrogate and all procedures were approved by the appropriate IRB. We found that either combined or separated T-scores for FTD patients did not exceed 70 and were not significantly different from control populations (Table 1).

**Table 1.** Suicidality on the PAI in 18 Patients With bvFTD and 3 Patients With svPPA.

Diagnosis	MDRS2 (SD)	Age (SD)	Suicidality T-score (SD)
bvFTD (n = 18)	123 (10)	57 (6)	51 (10)
PPA (n = 3)	110 (17)	63 (15)	53 (9)

Abbreviations: bvFTD, behavioral variant frontotemporal dementia; MDRS2, Mattis Dementia Rating Scale 2; PAI, Personality Assessment Inventory; PPA, primary progressive aphasia; SD, standard deviation; svPPA, semantic variant primary progressive aphasia.

The results of our analysis contrast with prior reports of elevated suicidality in patients with FTD. However, it is important to note differences in the patient populations examined. First, the number of patients we examined was small, due to the rarity of patients with known MAPT mutations in the early stages of the disease. The patients in our analysis had either early or prodromal symptoms and were found to have a clinical dementia rating score of 1 or less consistent with mild dementia. Zucca et al found that younger patients with a longer duration of illness were more likely to attempt suicide, and it is possible that since the population we examined had only mild dementia, they do not represent the same population that is at elevated risk for suicidality described by Zucca et al. Also, while we did not find elevated suicidal ideation in MAPT mutation carriers, C9orf72 expansion carriers (another genetic cause of FTD and amytrophic lateral sclerosis) have elevated risk of suicidal behaviors.^14^ This brings up the intriguing possibility that differences in the genetics of the FTD population examined may be directly related to different psychiatric symptoms including suicidality.^15^

The Zucca et al study demonstrates why it is imperative to evaluate all patients with FTD for suicidal thinking as this disorder, and many other dementing illnesses, are known to carry significant comorbidity for depression and increased risk of suicide. Further research is needed to better understand suicidal thinking and behaviors in FTD patients. In particular, it will be important to establish whether genetic factors or neuroanatomical factors play a role in the evolution of these symptoms in patients, as our data and others suggest it might.
